# The Primacy of Adipose Tissue Gene Expression and Plasma Lipidome in Cardiometabolic Disease in Persons With HIV

**DOI:** 10.1093/infdis/jiae532

**Published:** 2024-12-09

**Authors:** Samuel S Bailin, Siyuan Ma, Andrew S Perry, James G Terry, John Jeffrey Carr, Sangeeta Nair, Heidi J Silver, Mingjian Shi, Mona Mashayekhi, Jonathan A Kropski, Jane F Ferguson, Celestine N Wanjalla, Suman R Das, Ravi Shah, John R Koethe, Curtis L Gabriel

**Affiliations:** Department of Medicine, Division of Infectious Diseases, Vanderbilt University Medical Center, Nashville, Tennessee, USA; Department of Biostatistics, Vanderbilt University Medical Center, Nashville, Tennessee, USA; Department of Medicine, Division of Cardiology, Vanderbilt University Medical Center, Nashville, Tennessee, USA; Department of Radiology and Radiological Sciences, Vanderbilt University Medical Center, Nashville, Tennessee, USA; Department of Radiology and Radiological Sciences, Vanderbilt University Medical Center, Nashville, Tennessee, USA; Department of Radiology and Radiological Sciences, Vanderbilt University Medical Center, Nashville, Tennessee, USA; Department of Medicine, Division of Gastroenterology, Hepatology, and Nutrition, Vanderbilt University Medical Center, Nashville, Tennessee, USA; Veterans Health Administration, Tennessee Valley Healthcare System, Nashville, Tennessee, USA; Department of Biomedical Informatics, Vanderbilt University Medical Center, Nashville, Tennessee, USA; Department of Medicine, Division of Diabetes, Endocrinology, and Metabolism, Vanderbilt University Medical Center, Nashville, Tennessee, USA; Veterans Health Administration, Tennessee Valley Healthcare System, Nashville, Tennessee, USA; Department of Medicine, Division of Allergy, Pulmonary, and Critical Care Medicine, Vanderbilt University Medical Center, Nashville, Tennessee, USA; Department of Medicine, Division of Cardiology, Vanderbilt University Medical Center, Nashville, Tennessee, USA; Department of Medicine, Division of Infectious Diseases, Vanderbilt University Medical Center, Nashville, Tennessee, USA; Department of Medicine, Division of Infectious Diseases, Vanderbilt University Medical Center, Nashville, Tennessee, USA; Vanderbilt Translational and Clinical Cardiovascular Research Center, Department of Medicine, Division of Cardiology, Vanderbilt University Medical Center, Nashville, Tennessee, USA; Department of Medicine, Division of Infectious Diseases, Vanderbilt University Medical Center, Nashville, Tennessee, USA; Veterans Health Administration, Tennessee Valley Healthcare System, Nashville, Tennessee, USA; Department of Medicine, Division of Gastroenterology, Hepatology, and Nutrition, Vanderbilt University Medical Center, Nashville, Tennessee, USA

**Keywords:** adipose tissue, human immunodeficiency virus, transcriptome, lipidome, multiomics

## Abstract

**Background:**

Persons with HIV (PWH) on contemporary antiretroviral therapy (ART) are at elevated risk for developing age-related cardiometabolic diseases. We hypothesized that integrative analysis of cross-tissue, multimodal data from PWH could provide insight into molecular programming that defines cardiometabolic phenotypes in this high-risk group.

**Methods:**

We enrolled 93 PWH without diabetes who were virologically suppressed on contemporary ART and obtained measures of insulin resistance, glucose intolerance, and adiposity. We performed circulating lipidomics, proteomics, and metabolomics, as well as subcutaneous adipose tissue (SAT) bulk transcriptomics, and used multiomics factor analysis (MOFA) to perform integrative analyses of these datasets.

**Results:**

The median age was 43 years, median body mass index 30.8 kg/m^2^, 81% were male, and 56% were self-identified non-Hispanic White. We identified a specific MOFA factor associated with visceral adipose tissue volume (ρ = −0.43), homeostasis model assessment 2 insulin resistance score (ρ = −0.52), liver density (ρ = 0.43), and other cardiometabolic risk factors, which explained more variance in the SAT transcriptome and circulating lipidome compared with the circulating proteome and metabolome. Gene set enrichment analysis of this factor showed extracellular matrix and inflammatory pathways that primarily mapped to SAT myeloid cells and adipose progenitor cells using single-cell deconvolution. Lipidomic analysis showed that this factor was significantly enriched for triacylglycerol and diacylglycerol species.

**Conclusions:**

Our multiomic analysis demonstrated coordinated, multitissue molecular reprogramming in virologically suppressed PWH with elevated cardiometabolic disease risk. Longitudinal studies of PWH with assessments of adipose tissue and lipid handling are necessary to understand mechanisms of cardiometabolic disease in PWH.

**Clinical Trials Registration**. NCT04451980.

## Teaser

Multiomic analysis of the adipose tissue transcriptome, plasma metabolome, lipidome, and proteome demonstrated coordinated, multitissue molecular reprogramming in virologically suppressed PWH with elevated cardiometabolic disease risk, primarily reflected in the plasma lipidome and adipose tissue transcriptome.

## Graphical Abstract

**Graphical Abstract:**
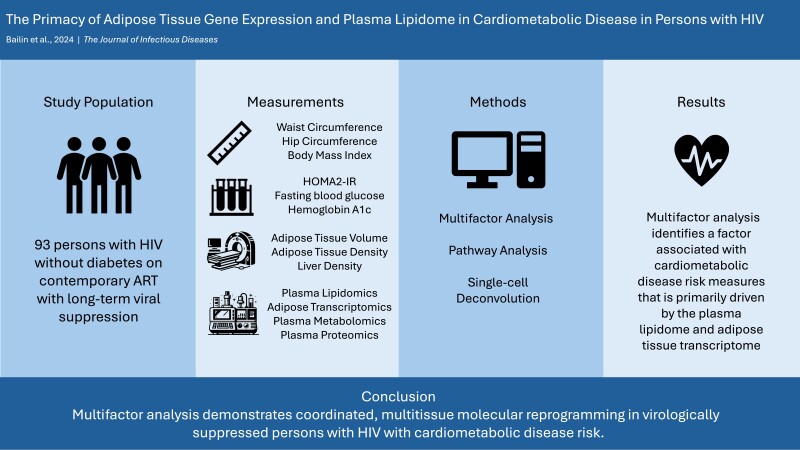

Persons with HIV (PWH) are at substantially higher risk for developing aging-related comorbidities compared to HIV-negative individuals [1], including cardiometabolic conditions such as cardiovascular disease, liver disease, diabetes mellitus, and dyslipidemia, which persists despite the suppression of plasma viremia on antiretroviral therapy (ART) [2–4]. As more than 50% of PWH are now over age 50 years [5], cardiometabolic disease-related morbidity and mortality is increasing and contributes to reduced quality of life and survival [6]. Therefore, identification of factors contributing to enhanced risk for age-related cardiometabolic diseases is critical for prevention and management strategies that improve the health of PWH.

Cardiometabolic disease in PWH is multifactorial and has been linked to persistent inflammation [7], HIV viral reservoirs, coinfections, gut microbiota dysbiosis and translocation, loss of immunoregulatory responses, and shunting of lipids from subcutaneous adipose tissue (SAT) to ectopic sites including liver and visceral adipose tissue (VAT) [8]. In the current era, these effects are compounded by rising rates of obesity and physical inactivity [9], which largely mirror long-term trends in the general population [10]. The interplay between genetics and environment, and their impact on metabolism, complicates efforts to identify factors contributing to heightened cardiometabolic risk in PWH.

High-throughput metabolomics and proteomics have enabled insight into how genetics, transcriptomics, and environmental factors interact and reshape the molecular landscape, contributing to phenotypic variance in PWH. Studies have reported alterations in circulating proteins, lipids, and metabolites linked to ART exposure [11], cardiometabolic disease [12–14], time to viral rebound [15], and frailty [16]. However, studies to date have primarily evaluated only one “omic” component in isolation, which limits inference of pathways spanning the multiome critical for cardiometabolic health. Additionally, adipose tissue has a critical role in regulating metabolism and has been mechanistically linked to cardiometabolic diseases. However, few analyses have included adipose tissue, which is frequently altered in PWH.

To address the need for a more comprehensive understanding of the global architecture of metabolic disease in PWH, we established a deeply phenotyped cohort of individuals with long-term virologic suppression and performed integrative, multiomic analyses of the circulating lipidome, metabolome, and proteome, as well as the SAT transcriptome. We found that circulating lipid species and the SAT transcriptome strongly associate with several interrelated measures of metabolism, including VAT volume, hepatic steatosis, and insulin resistance, to a much greater extent than the metabolome or proteome. Furthermore, we observed that the most heavily weighted SAT transcripts associated with cardiometabolic disease are primarily expressed in SAT macrophages. Together, these findings provide a more global view of the pathways underlying cardiometabolic disease in PWH and demonstrate the primacy of lipid metabolism and alterations in the adipose tissue immune compartment in explaining phenotypic variance across the spectrum of cardiometabolic disease.

## METHODS

### Study Cohort

Participants were part of the HIV Adipose Tissue Immunology and Metabolism (HATIM) study registered at clinicaltrials.gov (NCT04451980) [17]. We recruited individuals with a range of metabolic fitness from the Vanderbilt University Medical Center Comprehensive Care Clinic between August 2017 and March 2020. All participants were virologically suppressed for at least 1 year, on stable ART for at least 18 months, and had a CD4^+^ T cell count ≥350 cells/mm^3^ at enrollment. All participants fasted for at least 8 hours before plasma collection to measure fasting blood glucose (FBG) and insulin. The homeostasis model assessment 2 insulin resistance (HOMA2-IR) score was calculated using FBG and insulin measures. Body mass index (BMI), waist circumference, and hip circumference were obtained at the same visit. Only participants without diabetes and with SAT transcriptomic and plasma proteomic, lipidomic, and metabolomic data were included in this study. The study was approved by the Vanderbilt Institutional Review Board and all participants provided written informed consent.

### Adiposity Measurements

A Siemens Somatom Force multidetector computed tomography scanner was used for abdominal and chest imaging as previously published [18, 19]. Briefly, SAT and VAT volumes were measured at the L4–L5 vertebrae and imaging of the liver was obtained at the T12–L1 vertebrae. Liver radiodensity (lower density correlates with higher hepatic fat content) was quantified using Hounsfield units (HU) from 9 imaged regions, each section with 3 regions of interest. Pericardial adipose tissue volume was measured as previously described [20].

### Subcutaneous Adipose Tissue Collection and Bulk RNA Sequencing

Detailed methods have been previously published [21]. Briefly, SAT samples were obtained 3 cm right of the umbilicus with 2.1 mm blunt, side-ported liposuction catheter (Tulip Cell Friendly GEMS system Miller Harvester; Tulip Medical Products), rinsed in saline, placed in cryovials, and flash frozen in liquid nitrogen. Bulk RNA sequencing was performed on tissue samples as previously detailed (Gene Expression Omnibus [GEO] accession number GSE243957) [22]. Briefly, total RNA was extracted from cryopreserved samples and poly(A) messenger RNA (mRNA) enrichment was used before library construction. Samples underwent paired-end sequencing in 2 runs on the NovaSeq 6000 (Illumina). Read quality was assessed and mapped to the Human GENCODE release 34 using STAR version 2.7.3a and assigned to features using GENCODE version 34 annotations using FeatureCounts. edgeR was used to normalize gene counts to counts per million (cpm), and genes with cpm ≤ 1 in less than 50% of the samples were removed. The remaining genes were log_2_-transformed with a prior count of 2. The log cpm values were further corrected for batch effects with ComBat [23].

### Proteomic, Lipidomic, and Metabolomic Assays

Untargeted proteomics was performed on fasting plasma using the Olink Explore platform. Proteins were excluded if they did not pass the Olink quality control (n = 56), had a high degree of measures below the limit of detection (defined as proteins ≥90th percentile proportion of measures below the limit of detection; n = 295), or had a coefficient of variation > 30% (n = 191). Proteins were randomly selected for those with duplicate assays (n = 6). Proteins were standardized (mean 0, variance 1) prior to downstream analyses.

Untargeted lipidomics was performed on fasting plasma using liquid chromatography-high resolution tandem mass spectrometry (LC-HRMS) using established protocols [24]. Briefly, lipids were extracted using a modified Folch method. The extracted lipid was dried under nitrogen gas, reconstituted, and purified before injection with internal standards. A Q Exactive Orbitrap Mass Spectrometer with heated electrospray ionization coupled to a Dionex Ultimate 3000 UHPLC system (Thermo Fisher Scientific) in both positive and negative ion mode was used to perform LC-HRMS. The performance of spike and internal standards in each sample was used to validate the quality of acquisition. LipidMatch Flow software was used to annotate lipid species. Lipid species intensity was first log_2_ transformed, and then Z-scored (ie, mean-centered and standardized) for normalization. Missing values were imputed using one-fifth the minimum nonzero value for each lipid species separately. In total, 843 individual lipid species were analyzed including: fatty acyls, glycerolipids (diacylglycerols [DG], triacylglycerols [TG]), glycerophospholipids (cardiolipins, lyso-phosphatidylcholine, lyso-phosphatidylethanolamine, phosphatidylcholine, phosphatidylethanolamine, phosphatidylinositol, phosphatidylserines), prenol lipids, sphingolipids (ceramides, sphingomyelins), and sterol lipids.

Untargeted metabolomics was also performed as previously described [25, 26]. Proteins were precipitated using 8:1:1 acetonitrile: methanol: acetone ratio (Fisher Scientific). The supernatant was dried under a gentle stream of nitrogen. Samples were reconstituted with injection standards solution. LC-MS was performed on a Thermo Q-Exactive Orbitrap mass spectrometer equipped with a Dionex Ultra-High-Performance Liquid Chromatography system. Extraction and injection reproducibility was evaluated by calculating percent relative standard deviation of internal standard peak areas. Identification of features, deisotoping, feature alignment, and gap filling was completed with Mzmine 2. The data were searched against an internal retention time metabolite library at Southeast Center for Integrated Metabolomics.

### Analysis

Descriptive statistics for the cohort were summarized by median and interquartile values (continuous) and percentage (categorical). All normalized assay values were log_2_-transformed and scaled before further analysis. We used measures of adiposity (VAT, SAT, liver density), insulin resistance (HOMA-IR), and glucose intolerance (FBG and hemoglobin A1c) to understand how each biomarker relates to cardiometabolic phenotypic variance. We did not include cholesterol, triglyceride, low-density lipoprotein, or high-density lipoprotein values because these would likely correlate with lipidomic data. For single biomarker analysis, a Spearman correlation was performed for each biomarker and outcome measure. Species below a false discovery rate of 5% were shown with heatmaps using “Ward.D2” algorithm and visualized with ComplexHeatmap.

For the assay integration analysis, we utilized multiomics factor analysis (MOFA) implemented in the R package MOFA2 [27]. All assays (proteomic, SAT transcriptomic, metabolomic, lipidomic) were included. We fit the model with default parameters, including a total of 15 factors for the model to train on. For each omic layer, we calculated the variance explained by individual factors as well as the total variance explained by all 15 factors. To determine the factors most closely related to cardiometabolic measures, we performed Spearman correlations to examine associations of various metabolic and anthropometric measures with each of the derived MOFA factors. To evaluate the importance of each omics assay to this factor, we determined the variability explained by the factor for each omics assay. To evaluate the biological significance of gene/species weighting, we used ClusterProfileR to perform gene set enrichment analysis for the SAT transcriptomic assay with the model weights as the ranking using the Reactome Pathway Database. Pathway activity inference was performed using decoupleR. The PROGENy database was downloaded, and the ranked weighted gene list was input to run the multivariate linear model method. We performed lipid enrichment analysis with the lipid species weights using lipidr [28].

Finally, to deconvolute the bulk SAT transcriptome to cell-specific expression, we used 2 datasets. The first dataset is a previously published 162 552 single-cell RNA-sequencing (scRNA-seq) SAT stromal vascular fraction dataset (GSE198809) derived from PWH, some of whom overlap in this cohort (n = 38) [21]. The 400 highest positive- and negative-weighted genes were selected from the model using the get_weights function. The average expression by cell type for the selected genes was performed using the AverageExpression function implemented in the R package Seurat. The genes were scaled across cell types and visualized in a heatmap. Because scRNA-seq does not capture mature adipocytes, we also downloaded a single-nuclei RNA-sequencing dataset from HIV-negative persons that has been previously published (Single Cell Portal SCP1376) [29]. We filtered for only abdominal SAT tissue and performed the same analysis as above.

## RESULTS

### Plasma Metabolome, Lipidome, and Proteome and SAT Transcriptome Are Associated With Cardiometabolic Assessments

**Figure 1.**
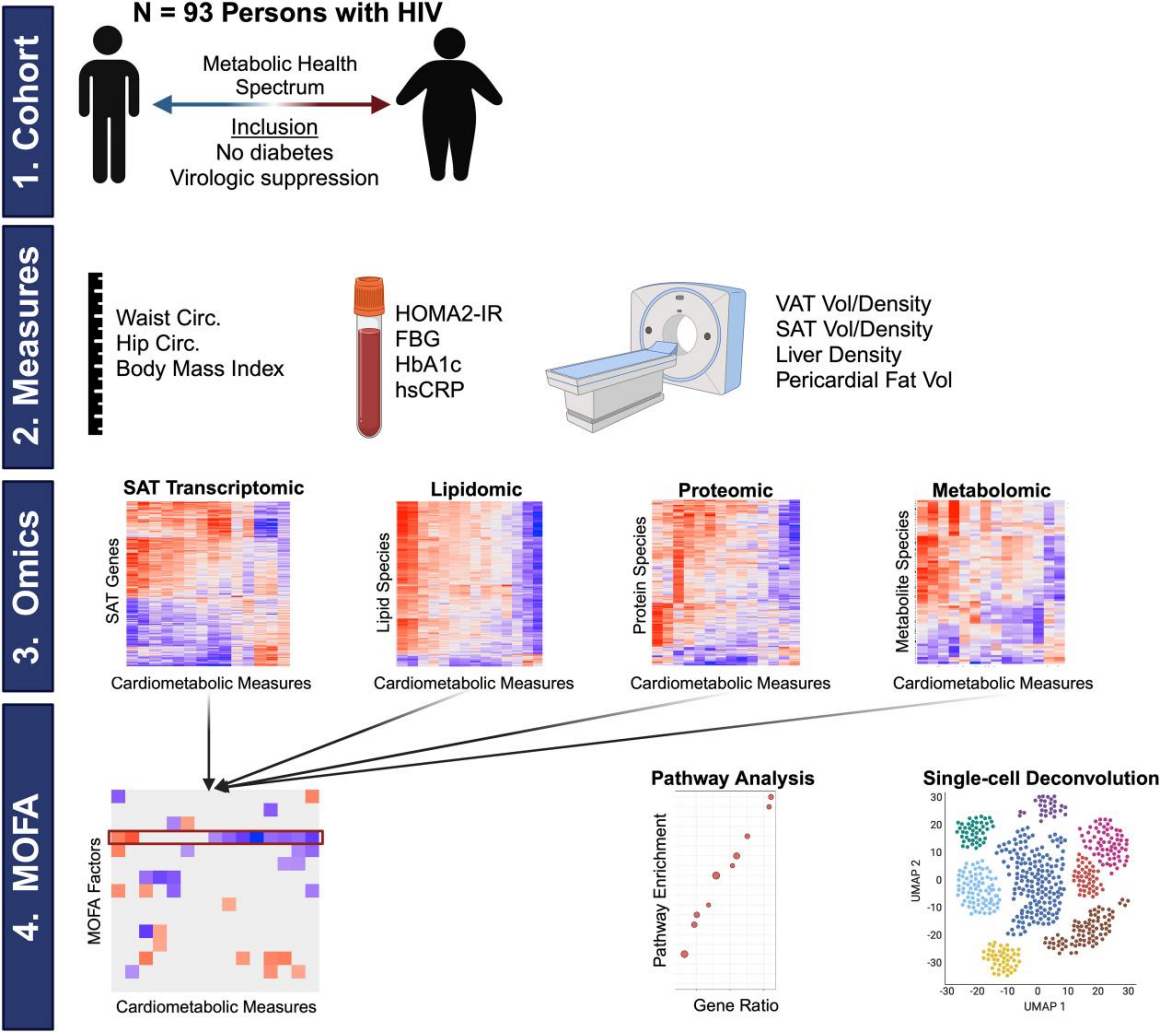
Study overview. A total of 93 persons with HIV virologically suppressed on contemporary antiretroviral therapy and without diabetes were included. Participants had several cardiometabolic assessments including waist circumference, hip circumference, body mass index, HOMA2-IR, fasting blood glucose, hemoglobin A1c, high-sensitivity C-reactive protein, and several morphometric measures by computed tomography including VAT volume and density, SAT volume and density, liver density (lower density correlates with higher hepatic fat content), and pericardial fat volume. Participants had whole-SAT transcriptomics and circulating lipidomics, metabolomics, and proteomics. Simple Spearman correlations were initially performed between cardiometabolic measures and individual assays. Then, we used MOFA to integrate across assays to identify a MOFA factor that was most closely associated with cardiometabolic features. We further analyzed this cardiometabolic factor including feature (assay) variance analysis, pathway and enrichment analysis, and single-cell RNA-sequencing deconvolution. Abbreviations: Circ, circumference; FBG, fasting blood glucose; HbA1c, hemoglobin A1c; HIV, human immunodeficiency virus; hsCRP, high-sensitivity C-reactive protein; HOMA2-IR, homeostasis model assessment 2 insulin resistance; MOFA, multiomics factor analysis; SAT, subcutaneous adipose tissue; UMAP, uniform manifold approximation and projection; VAT, visceral adipose tissue; Vol, volume. Generated with Biorender.

An overview of the cohort and analysis for this study is shown in Figure 1. Ninety-five individuals had a combination of lipidomic, metabolomic, proteomic, and SAT transcriptomic data. After multimodal quality control assessment and filtering, results from 93 individuals were carried forward for downstream analyses (Supplementary Figure 1*A*). The characteristics of the cohort are shown in Table 1. Median age was 43 years, median BMI 30.8 kg/m^2^, 81% were male, and 56% were self-identified non-Hispanic White. Participants had insulin resistance (median HOMA2-IR 2.35) and elevated VAT volume (median 153 cm^3^).

**Table 1.** Participant Characteristics

Characteristic	Total Cohort (n = 93)
Median Age, y (IQR)	43.0 (35.0, 53.0)
Median BMI, kg/m^2^ (IQR)	30.8 (28.6, 34.9)
Sex, female, No. (%)	18 (19)
Race, White, No. (%)	52 (56)
Median CD4 count, cells/mm^3^ (IQR)	803 (591, 986)
INSTI regimen, No. (%)	60 (65)
Median FBG, mg/dL (IQR)	96 (88, 110)
Median HbA1c, % (IQR)	5.4 (5.1, 5.5)
Median HOMA2-IR (IQR)	2.35 (1.49, 4.13)
Median VAT volume, cm^3^ (IQR)	153 (111, 186)
Median SAT volume, cm^3^ (IQR)	339 (244, 452)
Median Liver density, HU (IQR)	63 (58, 67)

Abbreviations: BMI, body mass index; FBG, fasting blood glucose; HbA1c, hemoglobin A1c; HOMA2-IR, homeostasis model assessment 2 insulin resistance; HU, Hounsfield unit; INSTI, integrase strand transfer inhibitor; IQR, interquartile range; SAT, subcutaneous adipose tissue; VAT, visceral adipose tissue.

**Figure 2.**
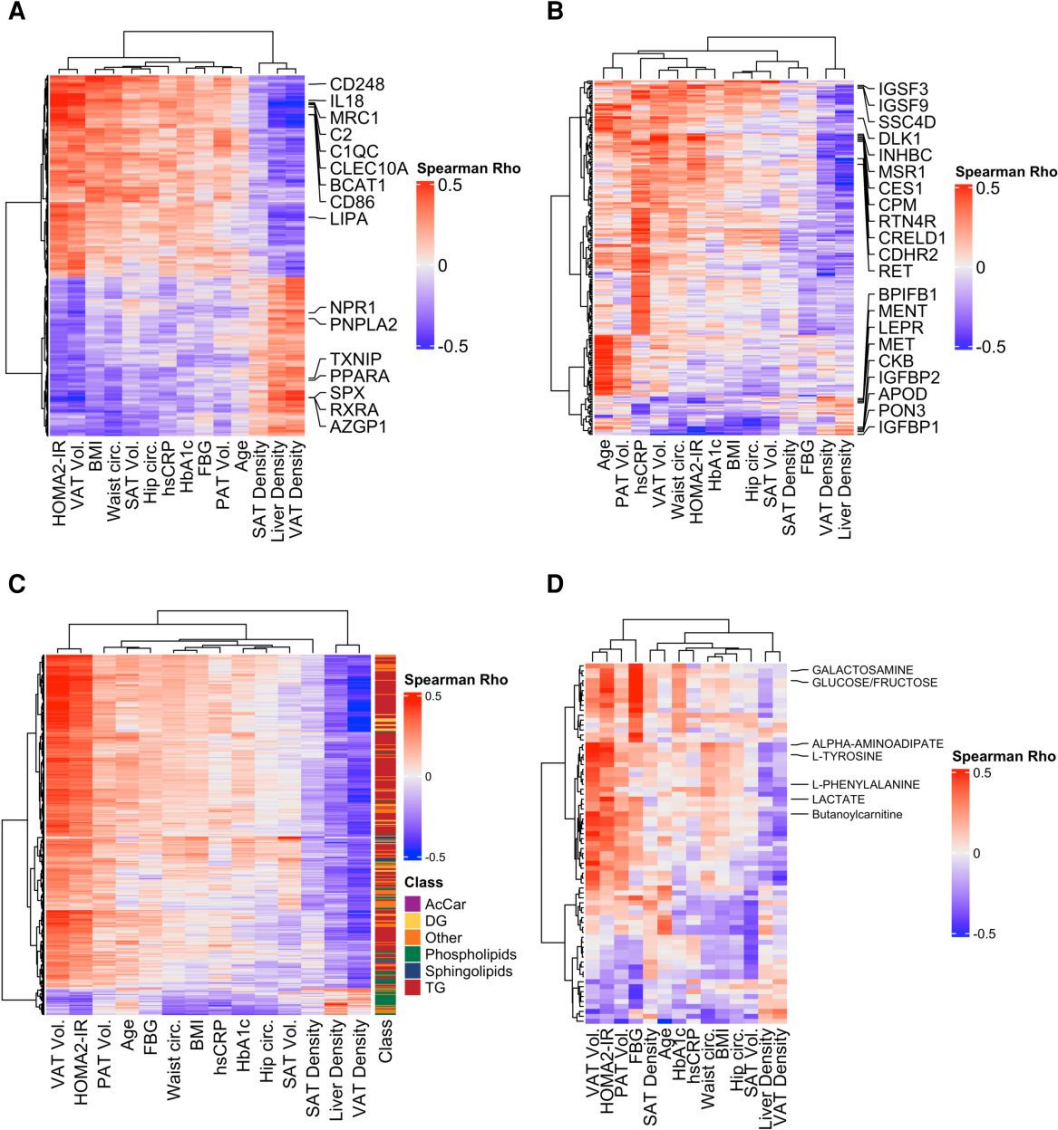
Cardiometabolic Measures are associated with species across different omic assays. *A*–*D*, Spearman correlation between the variable of interest (x-axis) and the individual measured gene or species (y-axis) plotted using the Ward D2 hierarchical clustering algorithm for both rows and columns. Select genes or species associated with insulin resistance or visceral adipose tissue volume were annotated. Only species at the cutoff of a false-discovery rate of 5% with any of the cardiometabolic measurements were included in the heatmap for (*A*) subcutaneous adipose tissue transcriptome, (*B*) circulating proteome, (*C*) circulating lipidome, (*D*) and circulating metabolome. Abbreviations: AcCar, acylcarnitine; BMI, body mass index; Circ., circumference; DG, diacylglycerol; FBG, fasting blood glucose; HbA1c, hemoglobin A1c; HOMA2-IR, homeostatic model assessment of insulin resistance 2; hsCRP, high-sensitivity C-reactive protein; PAT, pericardial adipose tissue; SAT, subcutaneous adipose tissue; TG, triacylglycerol; VAT, visceral adipose tissue; Vol., volume.

To examine the relationship of each metabolic profiling modality with measures of cardiometabolic disease and risk, we performed Spearman correlation between the assay and measured outcomes of interest, retaining those with a false discovery rate of 5%. Insulin resistance and VAT volume had similar associations with individual biomarker species, suggesting shared biology (Figure 2*A*–*D*). These unadjusted associations identified species that have been associated with insulin resistance and obesity for the protein (eg, *CES1, INHBC, RTN4R*) [30, 31], metabolite (α-aminoadipate, butyryl carnitine, tyrosine) [32, 33], and lipid (TG and DG species) assays [34]. Collectively, these data show that measurements across different classes of biomarkers are each associated with measures of adiposity and cardiometabolic disease.

### Integration of Omics Data Reveals a Factor That Correlates With Cardiometabolic Assessments

**Figure 3.**
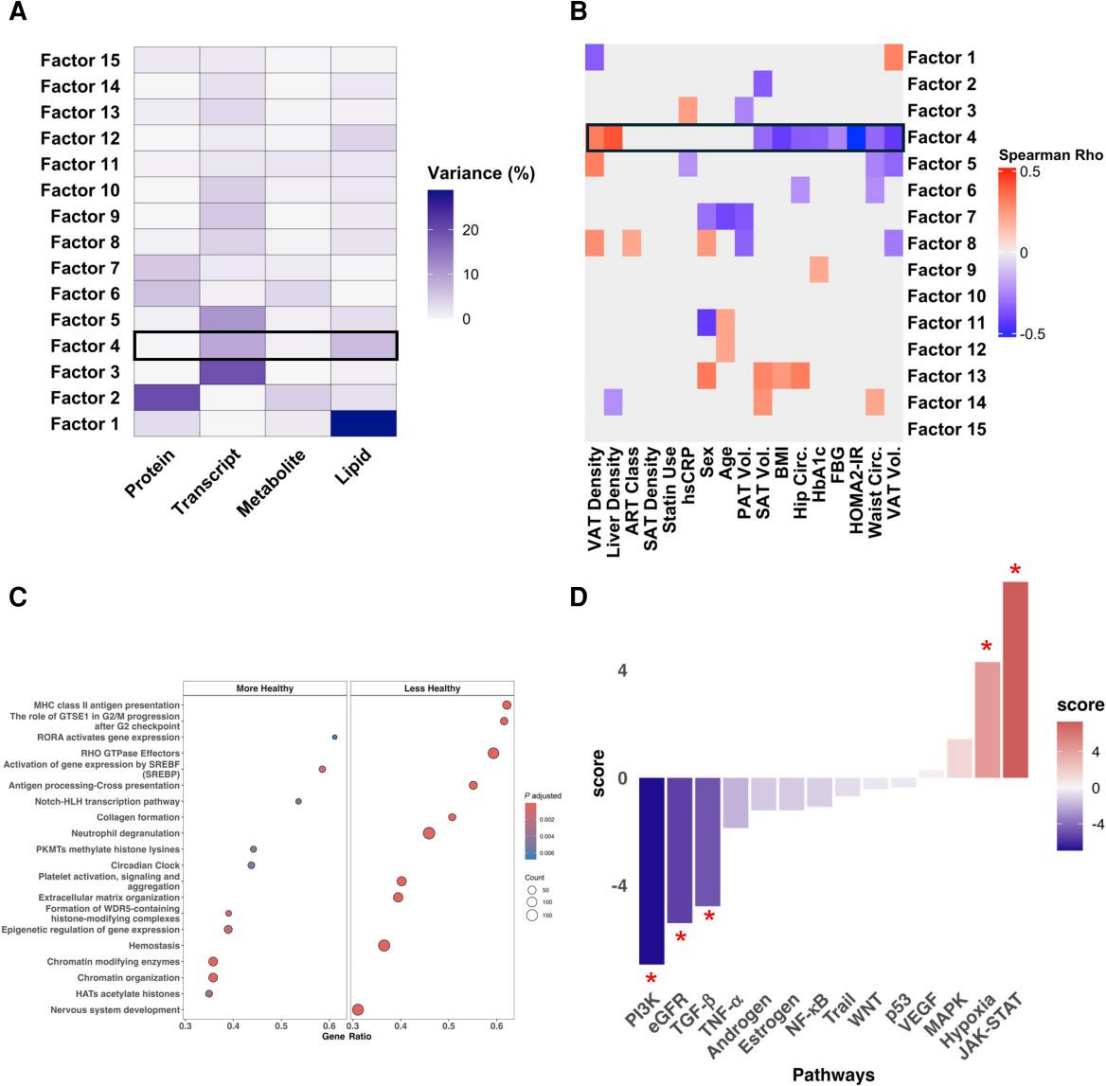
Integration of omics data reveals a factor that correlates with cardiometabolic assessments. *A*, Heatmap of assay type (x-axis) and MOFA factor (y-axis) showing the percent variance explained (shading). *B*, Heatmap of unadjusted Spearman correlation between cardiometabolic variable measures (x-axis) and MOFA factor (y-axis). Correlations with a *P* value < .05 are shaded in red or blue. Columns were plotted using the Ward D2 hierarchical clustering algorithm. *C*, Gene set enrichment analysis of SAT transcriptome with MOFA weights as the ranking using the Reactome database with gene ratio (x-axis) and pathway description (y-axis) split by positive (improved cardiometabolic risk factor measurements) and negative (worsened cardiometabolic risk factor measurements) enrichment. The dot size represents the number of genes in the pathway and the dot color represents the adjusted *P* value. *D*, Pathway activity inference using the PROGENy database shows changes in activity in positively weighted SAT genes compared with negatively weighted SAT genes. *P value < .05. Black border in (*A*) and (*B*) highlights factor 4, most closely associated with several cardiometabolic measures. Abbreviations: ART, antiretroviral therapy; BMI, body mass index; Circ., circumference; EGFR, epidermal growth factor receptor; FBG, fasting blood glucose; HbA1c, hemoglobin A1c; HOMA2-IR, homeostatic model assessment of insulin resistance 2; JAK-STAT, janus kinase-signal transducer and activator of transcription; MAPK, mitogen-activated protein kinase; MOFA, multiomics factor analysis; NF-κB, nuclear factor-κB; PI3K, phosphatidylinositol 3 kinase; SAT, subcutaneous adipose tissue; TGF-β, transforming growth factor-β; TNF-α, tumor necrosis factor-α; VAT, visceral adipose tissue; VEGF, vascular endothelial growth factor; Vol., volume; WNT, wingless/integrated.

We next evaluated whether integration across multiomic data could uncover coordinated changes across assays corresponding to cardiometabolic phenotypes in our at-risk cohort. We used MOFA to build an integrated model with 843 lipid species, 367 metabolites, 2384 proteins, and 14 969 SAT transcripts in 93 individuals with HIV and without diabetes [35]. We found 15 multiomic latent “factors,” that is, axes of coordinated, population-level covariations across the tissue molecular profiles; similar to single-omic principal components, each factor thus represents a major signal of covariation among multiple assays (Supplementary Figure 1*B* and 1*C*). While the first 3 factors represented omics assay-specific variations (Figure 3*A*), factor 4, hereafter termed the “cardiometabolic factor,” was associated with several cardiometabolic measures including HOMA2-IR, VAT volume, and liver steatosis (Figure 3*B*). These associations remain robust after adjusting for age, sex, BMI, and race for HOMA2-IR (ρ = −0.48, *P* = <.001), VAT volume (ρ = −0.32, *P* = .01), and liver steatosis (ρ = 0.33, *P* = .01). Statin use was not associated with any factor and ART class was modestly associated with factor 8 (ρ = 0.21, *P* = .05).

We next focused on the cardiometabolic factor to understand the structure and biologically relevant pathways to cardiometabolic diseases that are revealed by this factor. The cardiometabolic factor explained the most variability in SAT transcriptome (*R*^2^ = 8.85) and circulating lipidome (*R*^2^ = 6.64), with less variability explained for circulating proteome (*R*^2^ = 0.35) and metabolome (*R*^2^ = 0.91) data (Supplementary Material). We performed gene set enrichment analysis with Reactome using the SAT gene weights from the cardiometabolic factor and found enrichment of pathways related to regulation of cholesterol biosynthesis and transcriptional regulation of white adipocyte differentiation. In contrast, pathways related to neutrophil degranulation, extracellular matrix organization, collagen formation, and MHC class II antigen presentation were negatively enriched (associated with worse cardiometabolic measurements) (Figure 3*C* and Supplementary Material). Pathway activity inference analysis showed substantial pathway activity of phosphatidylinositol 3 kinase (PI3K), epidermal growth factor receptor (EGFR), and transforming growth factor-β (TGFβ) in the negatively weighted cardiometabolic factor compartment (Figure 3*D*). The SAT gene weighting and top enriched gene sets were largely unique to the cardiometabolic factor compared with other factors (Supplementary Figure 2*A* and 2*B*).

**Figure 4.**
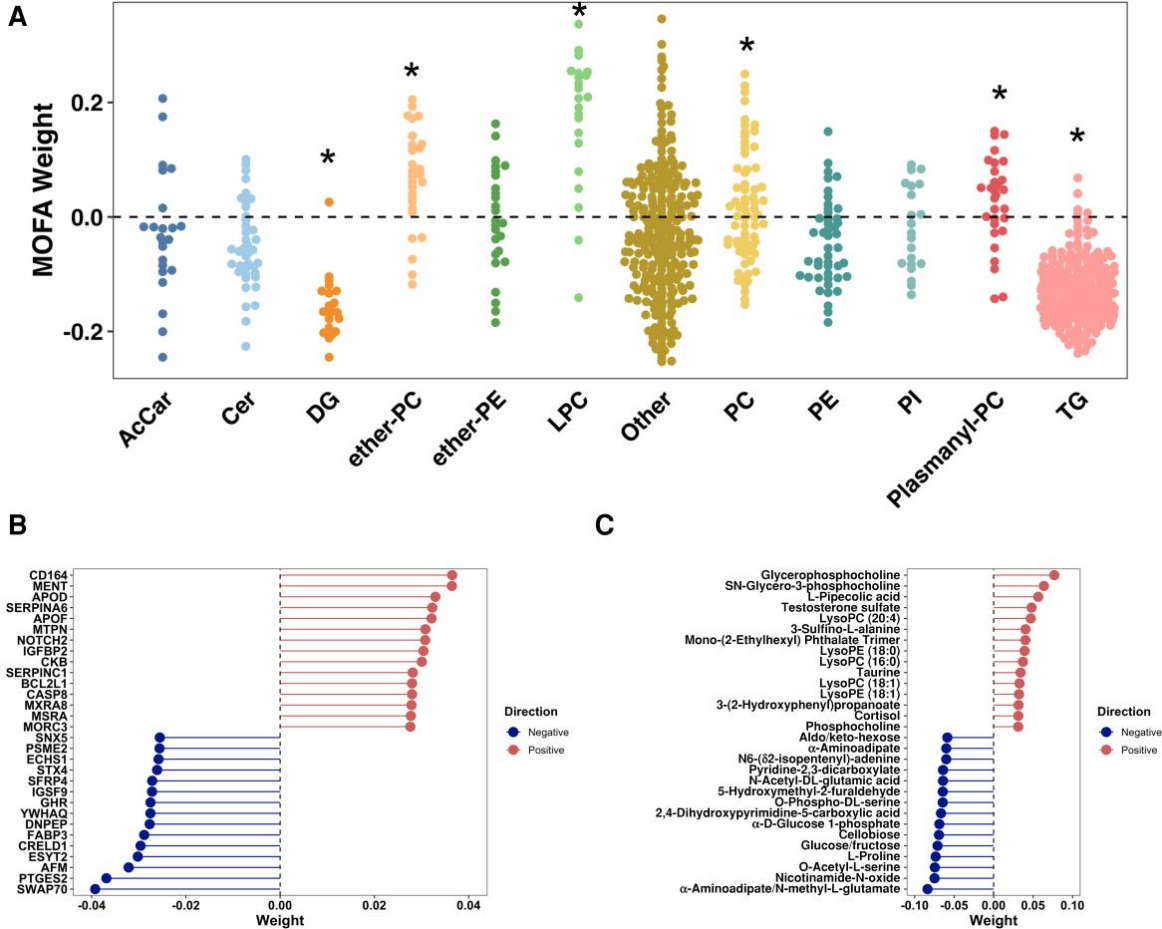
Diacylglycerol and triacylglycerol lipid species are heavily weighted in factor 4. *A*, Bee swarm plot with lipid class (x-axis) and MultiOmics Factor Analysis (MOFA) weighting from the cardiometabolic factor (y-axis). Negatively weighted factors are associated with worse cardiometabolic measurements. The asterisks denotes significant over- or underenrichment at a false-discovery rate of 5%. *B*, MOFA weighting for 15 highest and 15 lowest weighted circulating protein species from the cardiometabolic factor. The weighting is plotted on the x-axis while the protein species is plotted on the y-axis. The color corresponds to positive (red) and negative (blue) weighting. *C*, MOFA weighting for 15 highest and 15 lowest weighted circulating metabolite species from the cardiometabolic factor. The weighting is plotted on the x-axis while the metabolite species is plotted on the y-axis. The color corresponds to positive (red) and negative (blue) weighting. Abbreviations: AcCar, acylcarnitine; Cer, ceramide; DG, diacylglycerol; ether-PC, ether-linked phosphatidylcholine; ether-PE, ether-linked phosphatidylethanolamine; LPC, lysophosphatidylcholine; PC, phosphatidylcholine; PE, phosphatidylethanolamine; PI, phosphatidylinositol; plasmanyl-PC, plasmanyl-phosphatidylcholine; TG, triacylglycerol.

The lipid species were enriched in lyso-phosphatidylcholine and phosphatidylcholine in the cardiometabolic factor, which have been associated with decreased risk for developing insulin resistance and hepatic steatosis [11, 36]. In contrast, TG and DG lipid species were overrepresented in the negatively weighted cardiometabolic factor compartment (Figure 4*A*). There was no specific enrichment with degree of saturation by lipid class (Supplementary Figure 3). Protein and metabolite species had substantially lower weights in the cardiometabolic factor (Figure 4*B* and 4*C*). Thus, the critical role of adipose tissue in the storage and release of glycerol species as fatty acids links the abnormal transcriptomic architecture of SAT with the circulating lipidome.

### Deconvolution of SAT Transcriptional Architecture Shows a Major Role for Myeloid Cells

**Figure 5.**
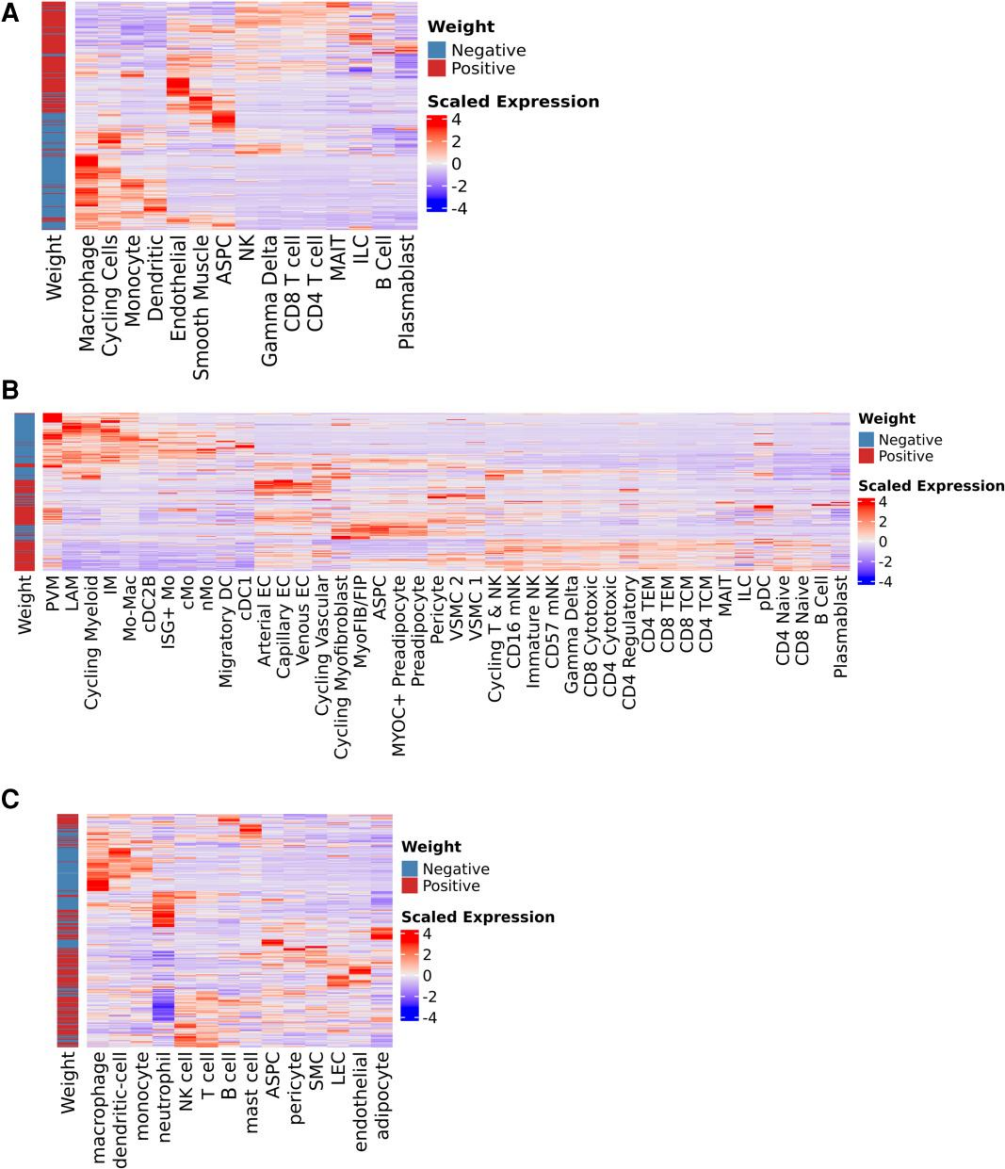
Highest weighted subcutaneous adipose tissue genes map to myeloid (negative) and stromal (positive) cells in single-cell/nuclei RNA-sequencing data. *A*–*C*, Heatmap of mean expression of the top 400 positively and negatively weighted genes from the MOFA factor 4 by cell type. The mean normalized expression for each cell type was scaled (y-axis) across cell types (x-axis) and plotted using the Ward D2 hierarchical clustering algorithm. Higher expression is in red while lower expression is in blue. The corresponding directionality of the MOFA gene weight (positive = red, negative = blue) is plotted on the left for (*A*) coarse cell type classification in the HATIM single-cell RNA-sequencing data set (n = 59), (*B*) fine cell type classification in the HATIM single-cell RNA-sequencing data set (n = 59), and (*C*) coarse cell type classification from single-nuclei RNA-sequencing data of subcutaneous adipose tissue (n = 13). Abbreviations: ASPC, adipose stem and progenitor cell; cDC1, conventional dendritic cell type 1; cDC2B, conventional dendritic cell type 2B; cMo, classical monocyte; DC, dendritic cell; EC, endothelial cell; FIP, fibroinflammatory progenitor; HATIM, Human Adipose Tissue Immunology and Metabolism; ILC, innate lymphoid cell; IM, intermediate macrophage; ISG, interferon-stimulated gene; LAM, lipid-associated macrophage; LEC, lymphatic endothelial cell; Mac, macrophage; MAIT, mucosal-associated invariant T cell; Mo, monocyte; MOFA, multiomics factor analysis; mNK, mature natural killer; MyoFIB, myofibroblast; nMo, nonclassical monocyte; NK, natural killer; pDC, plasmacytoid dendritic cell; PVM, perivascular macrophage; SMC, smooth muscle cell; TCM, T-cell central memory; TEM, T-cell effector memory; VSMC, vascular smooth muscle cell.

Given SAT bulk transcriptomics had substantial variance corresponding to the cardiometabolic factor, we leveraged existing SAT sc/snRNA-seq datasets to determine cell-type–specific expression patterns that may drive global transcriptional changes in SAT [21, 29]. In the SAT scRNA-seq from PWH, we observed that the top 200 negatively weighted genes in the cardiometabolic factor primarily mapped to myeloid populations (Figure 5*A*). In contrast, endothelial and smooth muscle cells primarily expressed positively weighted genes, while the adipose stem and progenitor cells and preadipocyte populations were mixed (Figure 5*B*). Using a snRNA-seq dataset from an HIV-negative cohort that includes mature adipocytes, mature adipocytes expressed primarily positively weighted genes (Figure 5*C*). In summary, negatively weighted genes in the cardiometabolic factor (associated with worse measures of cardiometabolic status), primarily map to SAT macrophage and immune populations while positively weighted genes (associated with improved measures of cardiometabolic status) primarily map to adipocyte and stromal populations. These data suggest that shifts in cell-type composition in part explain global expression programs in SAT that define cardiometabolic disease in PWH.

## DISCUSSION

PWH are at substantial risk for cardiometabolic diseases, which contributes to health disparities compared with HIV-negative individuals. Using circulating lipidomics, metabolomics, and proteomics, and SAT bulk transcriptomics across 93 PWH virologically suppressed on contemporary ART, we performed integrative multiomic analysis and found a factor associated with key measures of cardiometabolic health. This factor heavily weighted the SAT transcriptome and circulating lipidome assays, was unique compared to the other MOFA factors, and revealed biologically plausible genes, pathways, and activating programs in SAT associated with inflammation and extracellular matrix that largely mapped to SAT immune cells.

MOFA identified SAT transcriptomics as a major component of the cardiometabolic factor, consistent with prior studies showing substantial molecular adaptation with obesity, insulin resistance [37], and weight loss [38]. SAT has a pivotal role in systemic metabolism, regulating glucose homeostasis, free fatty acid storage and release, and secreting adipokines that have been linked to health and disease [39]. Numerous studies have identified adipose tissue inflammation as a seminal event in the pathogenesis of insulin resistance, predominantly mediated through adipose tissue macrophages and other immune cells [40, 41]. In the current study, we resolve negatively weighted transcripts in the cardiometabolic factor as deriving primarily from myeloid cell populations. A recent study deconvoluted cell types from bulk SAT transcriptomics and found that a lower proportion of adipocytes was associated with higher BMI [42]. Similarly, we find positively weighted transcripts in the cardiometabolic factor map to adipocytes. Additionally, many of the genes associated with cardiometabolic measures in that study were similarly highly negatively weighted (*MSC, UCHL1, ITIH5*) and positively weighted (*SPX, GPD1L, AZGP1, SLC4A3, SLC2A4*) in the current study [42]. While the association between SAT inflammation and fibrosis and thymidine analog treatment is well described [43], these agents are rarely used today. Contemporary ART may still have deleterious consequences on adipose tissue function and contribute to unique molecular signatures in PWH, but this is far less studied compared with thymidine analog exposure [44, 45]. Similarly, few studies have directly compared SAT from PWH and HIV-negative persons, although a recent study incorporating both PWH and HIV-negative persons without diabetes found some shared transcriptional changes with insulin resistance [46]. Future studies that include healthy HIV-negative individuals are necessary to define the HIV-specific versus general factors that shape SAT transcriptomics.

Circulating lipid species also had significant variance corresponding to the cardiometabolic factor, similar to a prior study that integrated lipidomic and metabolomic data [47]. The results align with a prior large prospective cohort study of PWH, which found a significant association of TG and DG species with incident diabetes, whereas phosphatidylcholine species showed an inverse association [14]. These relationships did not appear to differ by HIV serostatus. Several DG species in the current study were negatively weighted in the cardiometabolic factor (associated with worse cardiometabolic measurements) and likely accumulated in part due to excess free fatty acid liberation from adipocytes, which alters insulin signaling in muscle and liver [34]. In this study, we did not directly measure free fatty acids. However, the changes in SAT transcriptomics and circulating lipidomics identified by MOFA likely reflect shared metabolic reprogramming involving adipose tissue, liver, and muscle. This cardiometabolic factor is also associated with greater liver steatosis and VAT volume, which are risk factors for cardiometabolic disease and likely reflect ectopic lipid deposition in the context of disrupted adipose tissue lipid metabolism [48]. PWH have increased VAT volume compared with BMI-matched HIV-negative individuals [49], and are particularly prone to VAT volume expansion during the first 2 years after ART initiation [50]. Thus, future longitudinal studies enrolling individuals before and after ART initiation that study adipose tissue, liver, muscle, and circulating lipid species might provide insight into the changes that drive an accelerated cardiometabolic phenotype in PWH.

This study has several strengths. First, the cohort has detailed anthropometric and adiposity measurements using computed tomography, the gold standard for quantifying adipose tissue depots. Second, this cohort is at risk for developing cardiometabolic diseases and is representative of the population living with HIV in the Southern United States. Finally, this is the largest cohort of PWH on contemporary ART with concurrent SAT biopsies to date, which allows for a detailed analysis of the SAT transcriptomic architecture and its relationship with metabolic disease and circulating metabolite/lipid species. This study has several limitations. First, the cross-sectional design precludes an assessment of causality, and future longitudinal and mechanistic studies will be needed. Second, while the number of participants was the largest of its kind for a study using these methods, the overall sample size was relatively modest, which inherently limits the ability to explore models integrating across omics data. Third, most individuals in this study were overweight or had obesity. While this is representative of the population with HIV in the Southern United States, these results may not extrapolate to populations with a broader range of BMI or in other regions. Fourth, we did not perform sampling of other metabolically important tissues, including muscle and liver. Finally, we do not have an HIV-negative cohort with both SAT biopsies and plasma omics, so we are unable to determine which of the observed differences are specific to treated HIV versus generalizable to the general population.

In conclusion, in a cohort of PWH with virologic suppression on contemporary ART, we integrated data across multiple platforms to identify an architecture of cardiometabolic disease associated with a distinct SAT transcriptomic and plasma lipidomic profile. These data implicate dysregulation of SAT and dyslipidemia as central contributors of phenotypic variance for measures associated with cardiometabolic diseases. As such, further research to understand the mechanisms by which HIV and contemporary ART alters SAT composition and function is crucial to the development of future therapeutic interventions to reduce cardiometabolic morbidity and mortality in PWH.

## Supplementary Data

Supplementary materials are available at *The Journal of Infectious Diseases* online (http://jid.oxfordjournals.org/). Supplementary materials consist of data provided by the author that are published to benefit the reader. The posted materials are not copyedited. The contents of all supplementary data are the sole responsibility of the authors. Questions or messages regarding errors should be addressed to the author.

## Supplementary Material

jiae532_Supplementary_Data

## Notes

***Acknowledgment.*** We thank the participants of the HIV Adipose Tissue Immunology and Metabolism (HATIM) study cohort.

***Author contributions.*** S. S. B. and S. M. wrote the original draft. C. L. G., R. S., J. R. K., J. A. K., J. F. F., M. J., A. S. P., C. N. W., S. D., M. M., H. J. S., J. G. T., S. N., and J. J. C.. reviewed and edited the manuscript. S. S. B., C. L. G., C. N. W., R. S., and J. R. K. acquired funding. S. S. B., C. L. G., S. M., R. S., J. R. K., J. A. K., and J. F. F. contributed conceptualization. S. S. B., S. M., A. S. P., J. M., H. J. S., S. N., J. G. T., and J. J. C. performed statistical analysis.

***Disclaimer***. The funding authorities had no role in study design, data collection, analysis, interpretation, the decision to publish, or manuscript preparation.

***Data availability.*** Further information and request for resources and reagent should be directed to and will be fulfilled by the lead contact, S. B. This study did not generate new unique reagents or code. The bulk subcutaneous adipose tissue RNA-sequencing used for this analysis is available at GEO Series accession number GSE243957.

***Financial support.*** This work was supported by the National institute of Diabetes and Digestive and Kidney Diseases, National Institutes of Health (NIH; grant numbers R01DK112262 to J. R. K. and C. N. W., R01DK131529 to J. R. K., K23DK135414 to S. S. B. and J. R. K., K23DK135420 to C. L. G., J. R. K., and J. F. F., and R01DK117144 to J. F. F.); the National Heart, Lung and Blood Institute, NIH (grant number K23HL156759 to C. N. W. and J. R. K.), National Center for Research Resources, NIH Clinical and Translational Science Award (grant number UL1RR024975 to Vanderbilt); the National Institute of Allergy and Infectious Diseases, NIH (grant number P30AI110527 to the Tennessee Center for AIDS Research); the Doris Duke Charitable Foundation (grant number 2021193 to C. N. W. and J. R. K.); and the Burroughs Wellcome Fund (grant number 1021480 to C. N. W. and J. R. K.).
