# Supplementary material for: The Primacy of Adipose Tissue Gene Expression and Plasma Lipidome in Cardiometabolic Disease in Persons With HIV
Source: J Infect Dis. 2024 Dec 9;231(2):e407–18. doi: 10.1093/infdis/jiae532 (PMC11841643; doi:10.1093/infdis/jiae532)
Supplement: jiae532_Supplementary_Data [file jiae532_supplementary_data.zip › supplemental material legends.docx]

**Supplemental Figure 1. (A)** Overview of missingness for each assay (subcutaneous adipose tissue transcriptome, circulating proteome, circulating metabolome, and circulating lipidome) for 93 participants included in the analysis. **(B)** Spearman’s correlation matrix between 15 factors from the Multi-Omics Factor Analysis (MOFA) model. Circle size is proportional to the Spearman’s rho. (**C**) Total explained variance (percent) for each assay (x-axis) across all 15 MOFA factors.

**Supplemental Figure 2. (A)** Upset plot with the top 50 reactome pathways based on adjusted p-value with intersection size on the x-axis and set size on the y-axis showing the overlap of top pathways from factor 4 with the other factors (**B**) Principal component analysis (PCA) using the subcutaneous adipose tissue transcriptome weights for each model. PC 1 (x-axis) and PC 2 (y-axis) are plotted for each factor from the model.

**Supplemental Figure 3.** Log–fold change of enrichment in the cardiometabolic factor (factor 4) plotted by total chain unsaturation (x-axis) and total chain length(y-axis) for each major lipid species. Abbreviation: AcCar, Acylcarnitine; Cer, ceramide; DG, diacylglycerol; LPC, lyso-phosphatidylcholine; PC, phosphatidylcholine; PE, phosphatidylethanolamine; PI, phosphatidylinositol; TG, triacylglycerol.
