# Supplementary figures and images for: The Primacy of Adipose Tissue Gene Expression and Plasma Lipidome in Cardiometabolic Disease in Persons With HIV
Source: J Infect Dis. 2024 Dec 9;231(2):e407–18. doi: 10.1093/infdis/jiae532 (PMC11841643; doi:10.1093/infdis/jiae532)

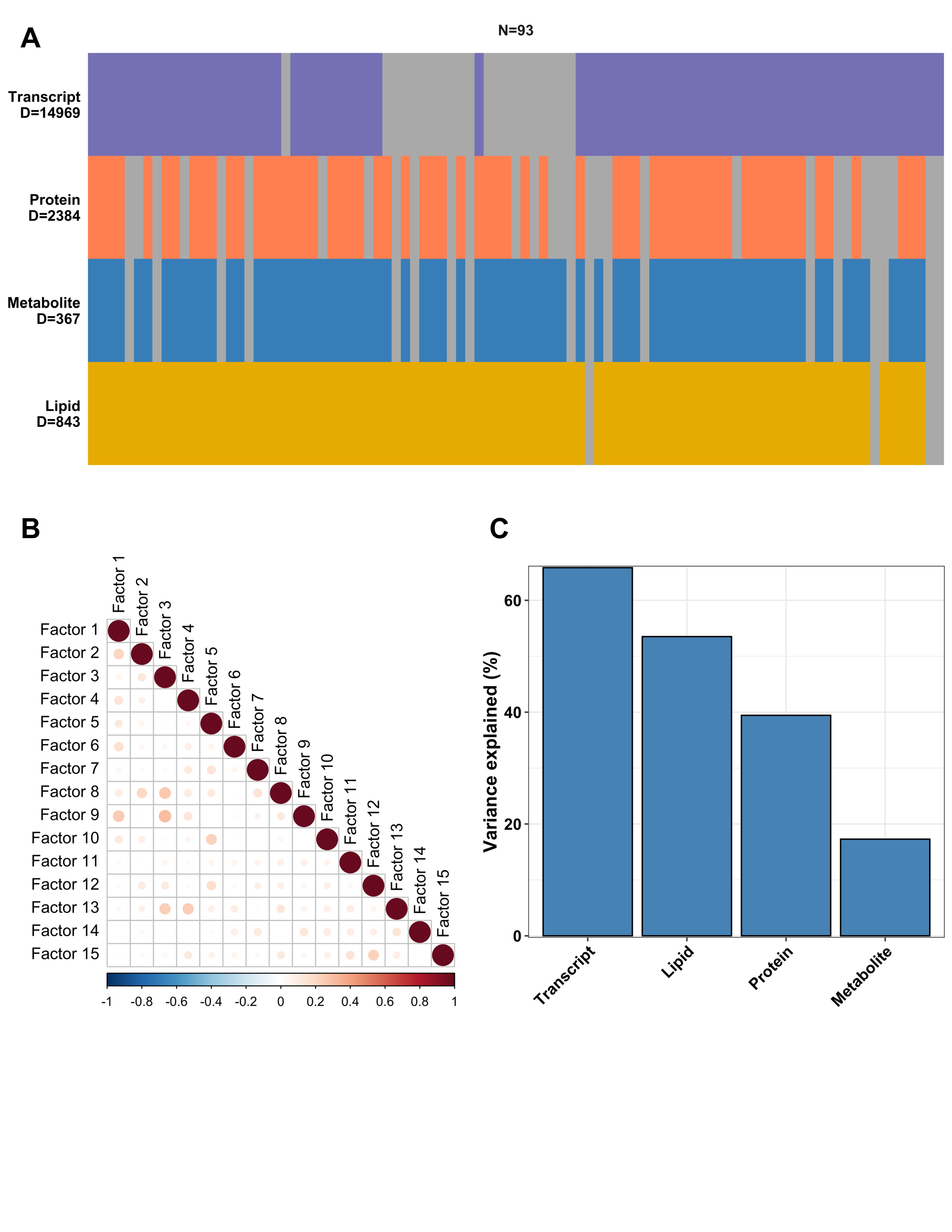

Supplement: jiae532_Supplementary_Data [file jiae532_supplementary_data.zip › Bailin_SF1.tif]

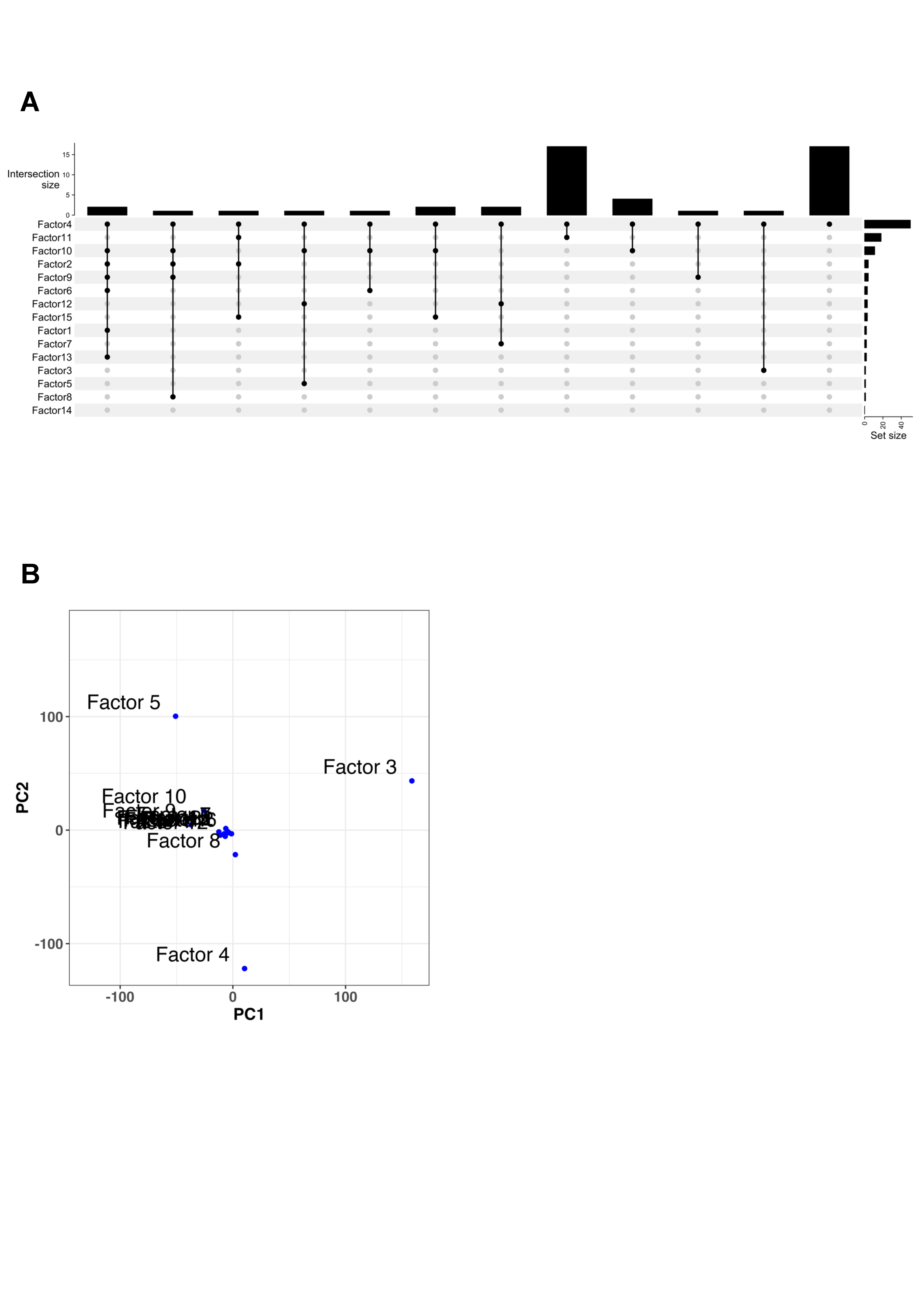

Supplement: jiae532_Supplementary_Data [file jiae532_supplementary_data.zip › Bailin_SF2.tif]

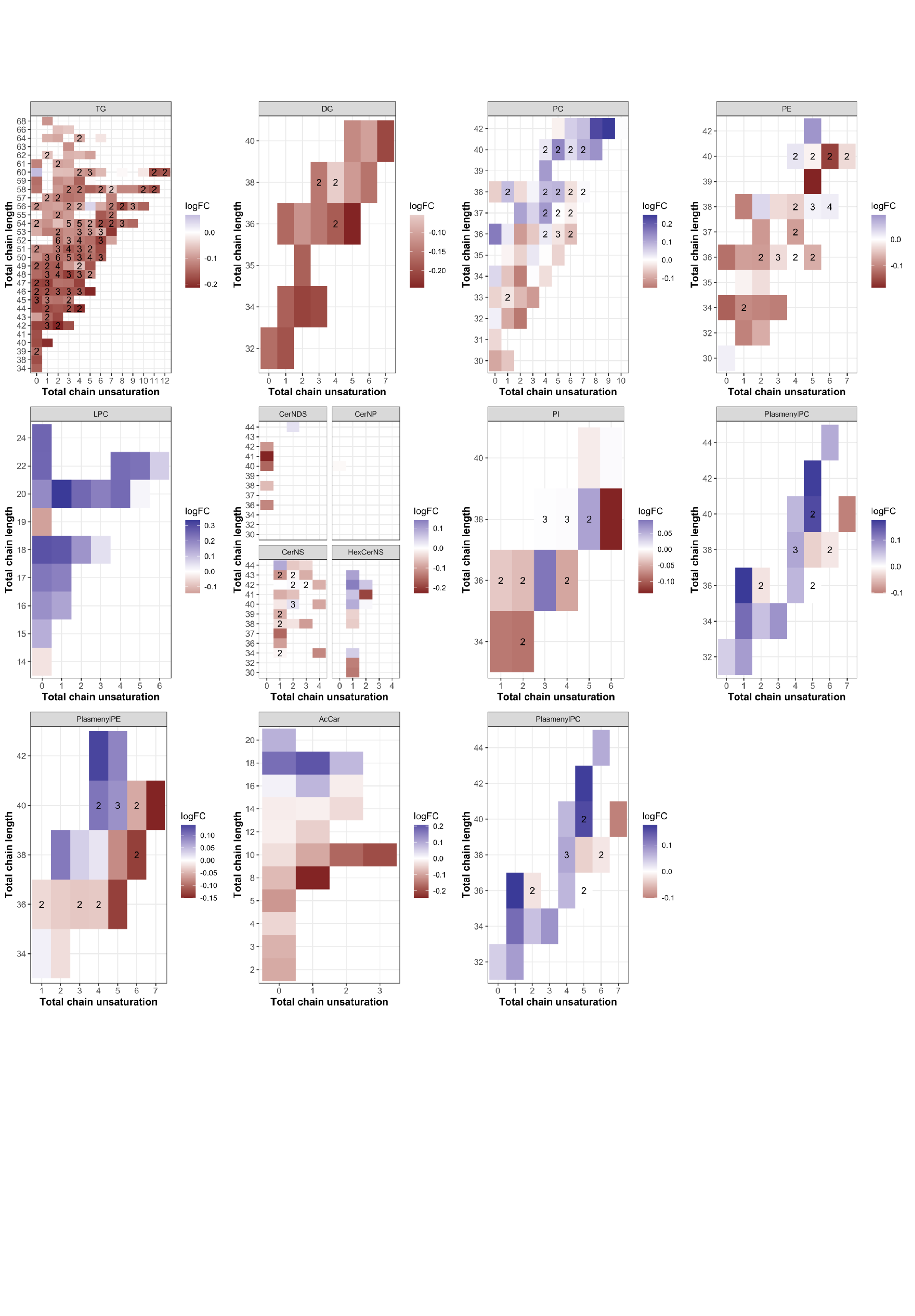

Supplement: jiae532_Supplementary_Data [file jiae532_supplementary_data.zip › Bailin_SF3.tif]
